# Subsurface seawater methylmercury maximum explains biotic mercury concentrations in the Canadian Arctic

**DOI:** 10.1038/s41598-018-32760-0

**Published:** 2018-09-27

**Authors:** Kang Wang, Kathleen M. Munson, Alexis Beaupré-Laperrière, Alfonso Mucci, Robie W. Macdonald, Feiyue Wang

**Affiliations:** 1 Centre for Earth Observation Science, and Department of Environment and Geography, University of Manitoba, Winnipeg, Manitoba R3T 2N2 Canada; 2 GEOTOP, and Department of Earth and Planetary Sciences, McGill University, Montreal, Quebec H3A 0E8 Canada; 3 Institute of Ocean Sciences, Department of Fisheries and Oceans, Sidney, British Columbia V8L 4B2 Canada

## Abstract

Mercury (Hg) is a contaminant of major concern in Arctic marine ecosystems. Decades of Hg observations in marine biota from across the Canadian Arctic show generally higher concentrations in the west than in the east. Various hypotheses have attributed this longitudinal biotic Hg gradient to regional differences in atmospheric or terrestrial inputs of inorganic Hg, but it is methylmercury (MeHg) that accumulates and biomagnifies in marine biota. Here, we present high-resolution vertical profiles of total Hg and MeHg in seawater along a transect from the Canada Basin, across the Canadian Arctic Archipelago (CAA) and Baffin Bay, and into the Labrador Sea. Total Hg concentrations are lower in the western Arctic, opposing the biotic Hg distributions. In contrast, MeHg exhibits a distinctive subsurface maximum at shallow depths of 100–300 m, with its peak concentration decreasing eastwards. As this subsurface MeHg maximum lies within the habitat of zooplankton and other lower trophic-level biota, biological uptake of subsurface MeHg and subsequent biomagnification readily explains the biotic Hg concentration gradient. Understanding the risk of MeHg to the Arctic marine ecosystem and Indigenous Peoples will thus require an elucidation of the processes that generate and maintain this subsurface MeHg maximum.

## Introduction

**Figure 1:**
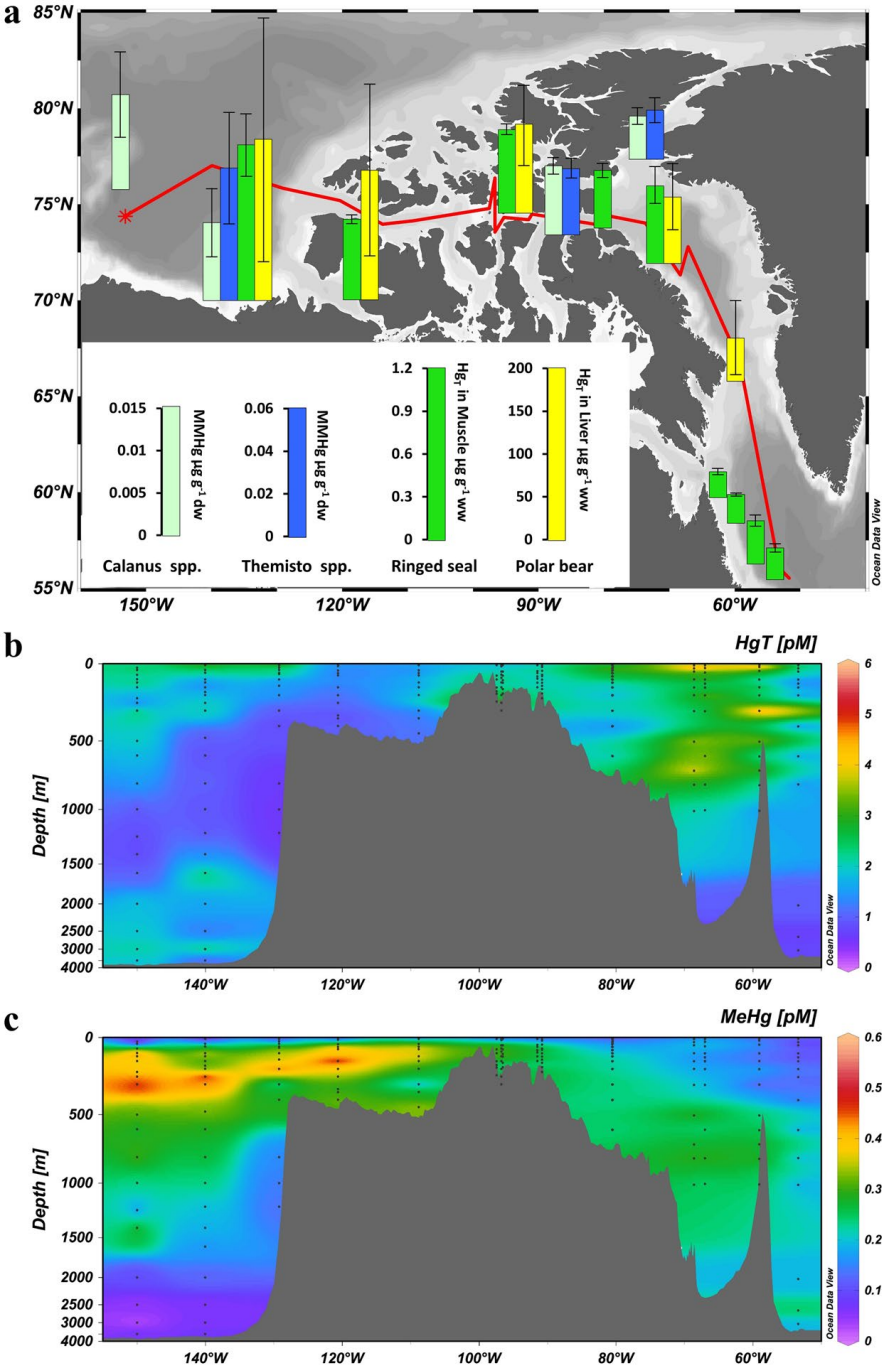
Mercury concentrations in the marine food web and seawater across the Canadian Arctic and Labrador Sea. (**a**) Map showing Hg concentrations in the marine food web, and seawater sampling sites; (**b**) distribution of total Hg (Hg_T_); and (**c**) methylmercury (MeHg) in seawater along a longitudinal (west-to-east) section. The bar charts in (**a**) show mean concentrations ± one standard deviation of monomethylmercury (MMHg) in *Calanus* spp. and *Themisto* spp. collected from 1998 to 2012^6^, total Hg (Hg_T_) in muscle of adult ringed seals collected in 2007 and 2011^3,5^, and Hg_T_ in liver of polar bears collected from 2005 to 2008^3^. The base map with bathymetry was created using Ocean Data View (version 4.0)^40^.

Monitoring data collected during the past four decades have shown Hg concentrations in Canadian Arctic marine mammals (e.g., beluga whales, ringed seals, polar bears) to be highly elevated, frequently exceeding toxicity thresholds^1,2^. This has raised major concerns over the health of marine mammals and Indigenous Peoples whose traditional diets include marine mammal tissues. Mercury concentrations in marine biota are generally higher in the Beaufort Sea and western Canadian Arctic Archipelago (CAA) than in the eastern CAA and Baffin Bay^1–3^. This longitudinal gradient is not limited to apex predators^4,5^, but extends to organisms at lower trophic levels such as zooplankton (e.g., *Themisto* spp., *Calanus* spp.)^6^ (Fig. 1a). Whereas regional variations in top predator Hg concentrations may be linked to feeding behavior and dietary preference^5^, observed spatial patterns persist after adjustments are made to account for trophic position^3^.

Extensive efforts have been made to identify factors that control the spatial trends in marine biota and to develop appropriate mitigation strategies to reduce biotic Hg concentrations. Most hypotheses attribute higher marine biotic Hg concentrations in the western Canadian Arctic to elevated inputs of inorganic Hg to these regions. These inputs include (1) atmospheric deposition of anthropogenic Hg from Asian sources^3^, which is enhanced locally by atmospheric mercury depletion events (AMDEs) during polar sunrise^7^; (2) riverine Hg input from the Mackenzie River^8,9^, which may be enhanced by tundra uptake of atmospheric elemental Hg^10^ [and permafrost thawing^11^; and (3) a naturally high geological background of Hg^12^. These inorganic Hg-based hypotheses do not account for the fact that it is methylmercury (MeHg), not inorganic Hg, that accumulates and biomagnifies in marine biota^2^. The discovery of a subsurface MeHg enrichment in global oceans^13–16^ suggests that seawater MeHg may play a more important role in determining marine biotic Hg concentrations^17,18^, especially in regions such as the Beaufort Sea^15^ and the central Arctic Ocean^16^ where the maximum MeHg concentration was observed at shallow depths.

During the 2015 Canadian Arctic GEOTRACES cruises (July 14–September 16, 2015) aboard the Canadian Research Icebreaker *CCGS Amundsen*, we measured high-resolution vertical profiles of total mercury (Hg_T_) (Fig. 1b) in unfiltered seawater, along a 5200-km transect (150°–53° W) from the Canada Basin in the west, through CAA to Baffin Bay in the east, reaching the Labrador Sea in the North Atlantic Ocean (Figs 1a, S1). Total Hg concentrations show a distinctive longitudinal gradient along the transect (Fig. 1b, Table 1), with concentrations increasing from the Canada Basin and the western CAA (1.76 ± 1.15 pM) to the eastern CAA and Baffin Bay (2.62 ± 1.97 pM). These results are comparable to the limited Hg_T_ dataset reported for Canadian Arctic waters^15,19,20^. The lowest concentrations were found in the Labrador Sea (0.65 ± 0.18 pM), slightly higher than those (0.44 ± 0.10 pM, 0.25–0.67 pM) measured in June 2014 in a similar area^21^.

**Table 1:** Concentrations of total Hg (Hg_T_) and methylmercury (MeHg) in seawater from the Canadian Arctic and Labrador Sea.

Regions	Stations	Depth	Hg_T_ (pM)	MeHg (pM)
Canada Basin, Beaufort Sea, and Western CAA	CB1–4; CAA6–9	0–500 m	1.90 ± 1.25, 0.73–8.55, n = 77	0.30 ± 0.14, 0.02–0.56, n = 77
		Full depth	1.76 ± 1.15, 0.55–8.55, n = 101	0.27 ± 0.14, 0.02–0.56, n = 100
Eastern CAA and Baffin Bay	CAA1–5; BB1–3	0–500 m	2.60 ± 2.06, 0.80–12.35, n = 78	0.19 ± 0.08, 0.04–0.44, n = 78
		Full depth	2.62 ± 1.97, 0.80–12.35, n = 93	0.20 ± 0.09, 0.04–0.44, n = 93
Labrador Sea	K1	0–500 m	0.62 ± 0.19, 0.30–0.92, n = 9	0.09 ± 0.04, 0.03–0.12, n = 9
		Full depth	0.65 ± 0.18, 0.30–0.95, n = 14	0.12 ± 0.06, 0.03–0.24, n = 15

The low seawater Hg_T_ concentrations in the Canada Basin, to the west, contrast with expectations based on the hypothesized elevated atmospheric and riverine inputs of Hg into this region. Whereas AMDEs do result in high springtime deposition, the low Canada Basin Hg_T_ concentrations are consistent with findings that most AMDEs occur in coastal regions and most of the deposited Hg is re-emitted to the atmosphere before snow melts^22^, thus limiting its transfer into the ocean. Likewise, Hg transported by rivers, possibly enhanced by tundra uptake, permafrost thawing, or geological enrichment, is likely deposited with sediment in coastal areas^23^ or escapes rapidly from the river plume to the atmosphere^9^. Furthermore, because MeHg accounts for <1% of the Hg_T_ in the Mackenzie River water^8,23^, and the atmospheric input of Hg is predominantly inorganic^22,24,25^, the Hg delivered to Canada Basin waters requires transformation to MeHg, the biomagnifying Hg species, to account for observed higher biotic Hg concentrations. Therefore, elevated input of inorganic Hg in the western relative to the eastern Arctic does not provide, by itself, a plausible mechanism to explain higher biotic Hg concentrations in the western Canadian Arctic (Fig. 1b).

Concentrations of MeHg (0.23 ± 0.12 pM, 0.02 to 0.56 pM), measured during the 2015 Canadian Arctic GEOTRACES cruises (Fig. 1c), are comparable to values reported in previous studies^15,19,20^ and show an overall decoupling from Hg_T_ distributions in the water column (Fig. 1c). The improved sampling resolution reveals distinctive vertical and longitudinal variations along the transect. Vertically, MeHg concentrations are lowest at the surface, increase with depth to a subsurface maximum, and subsequently decrease towards the bottom. Longitudinally, the subsurface MeHg peak value is highest (~0.5 pM) in the western part of the section and decreases to ~0.2 pM over the Barrow Strait sill into the eastern CAA, eventually dropping to ~0.1 pM in the Labrador Sea (Fig. 1c, Table 1). The depth of the subsurface MeHg maximum varies from west to east: MeHg peaks at depths of ~300 m at the westernmost station in the Canada Basin and shoals progressively eastward to ~100 m in the western CAA. Farther east, the subsurface MeHg peak remains at ~100 m in the eastern CAA and Baffin Bay, but deepens to ~200 m in the Labrador Sea.

Regional differences in polar bear hair Hg concentrations between the Beaufort Sea and Hudson Bay were tentatively attributed to regional differences in seawater MeHg concentrations that resulted in different degrees of bioaccumulation^18^, but high-resolution (vertical and horizontal) water-column MeHg concentration data were not available at that time to support this hypothesis. The distribution of the subsurface MeHg peak along our transect directly links the spatial distribution of aqueous MeHg concentrations to biotic uptake (Fig. 1a,c).

Enrichment of MeHg in the subsurface water column (300–1000 m) is a common feature of many ocean basins^13,14^. A notable difference is that the subsurface MeHg peak occurs at a much shallower depth in the western Canadian Arctic (100–300 m), in agreement with recent reports from the Beaufort Sea^15^ and the central Arctic Ocean^16^. This MeHg maximum occurs at shallow depths that are just below the surface productive layer (see Fig. S2), which may enhance MeHg availability to organisms at the base of the marine food webs^16^. Phytoplankton are known to bioconcentrate MeHg from seawater^26^, and zooplankton bioaccumulate it directly from seawater and by trophic transfer through their diet^27,28^; as a result, higher phytoplankton and zooplankton MeHg concentrations have been linked to higher seawater MeHg concentrations^29^. Among the three most important herbivores in Arctic waters, *Calanus hyperboreus* and *C. finmarchicus* are concentrated in shallow water (<300 m) except during winter, whereas *C. glacialis* spend all life stages in the top 300 m^30,31^. The amphipod consumers of these *Calanus* species, *Themisto* spp., also inhabit shallow waters^32^, as does Arctic cod (*Boreogadus saida*,<500 m), key species in the Arctic marine food web^33^. Given that the MeHg-enriched waters lie within the main habitat of low trophic level marine biota in these waters, spatial variations in MeHg concentrations within the subsurface zone can readily explain the higher biotic Hg concentrations in the western compared to the eastern regions of the section.

Therefore, to understand what controls Arctic biotic Hg distributions and predict future conditions, characterization of atmospheric and terrestrial sources of inorganic Hg inputs to the Arctic Ocean is not sufficient. Detailed investigations will be required to identify processes controlling the production and loss of MeHg associated with the upper halocline waters of the western Arctic Ocean and how these processes respond to the changing climate. The subsurface seawater MeHg maximum in the oceans is typically attributed to *in situ* MeHg production associated with organic matter remineralization^13–16^. In the central Arctic Ocean, Heimbürger *et al*.^16^ suggested that sinking particles are slowed down at the shallow pycnocline where they undergo remineralization and stimulate *in situ* MeHg production. It remains unclear what microbial or abiotic processes are responsible for Hg methylation at such shallow depths where dissolved oxygen is well above 75% of the saturation value (Fig. S2). Alternatively, the MeHg maximum in the upper halocline in the western Canadian Arctic could be supported by isopycnal transport, along with the metabolite-enriched upper halocline waters, from sediments of the productive Chukchi and Beaufort Shelves^34^. Understanding the risk of MeHg to the Arctic marine ecosystem and Indigenous Peoples will thus require an elucidation of the processes that generate and maintain the subsurface seawater MeHg maximum.

## Methods

Seawater sampling and analyses were carried out following ultraclean techniques recommended for the GEOTRACES program^35,36^. Seawater was collected onboard the Canadian Research Icebreaker *CCGS Amundsen* in pre-cleaned, 12-L Teflon-coated Go-Flo bottles mounted on a Trace Metal-Clean Rosette System. Following rosette retrieval, the Go-Flo bottles were promptly moved to a clean laboratory van where seawater for Hg_T_ and MeHg analyses was collected into pre-cleaned, 250-mL amber glass bottles^37^. Immediately after collection, the seawater samples were acidified with 0.5% (v:v) ultraclean acid (CMOS grade JT Baker HCl for Hg_T_, and trace metal clean grade Fisher Scientific H_2_SO_4_ for MeHg) and stored at 4 °C until analysis. The acidification breaks down dimethylmercury (DMHg) to monomethylmercury (MMHg)^38^ and, thus, the MeHg reported herein represents the sum of MMHg and DMHg.

Within 48 hr of sampling, Hg_T_ was analyzed in the Portable *In-situ* Laboratory for Mercury Speciation (PILMS) onboard the icebreaker (http://www.amundsen.ulaval.ca/capacity/portable-insitu-lab-mercury-speciation.php). The analysis was carried out on a Tekran 2600 Hg analyzer following U.S EPA Method 1631, which involves BrCl oxidation, SnCl_2_ reduction, gold trap pre-concentration and measurement by cold vapor atomic fluorescence spectrometry (CVAFS). Water samples were analyzed for MeHg at the PILMS or at the Ultra-Clean Trace Elements Laboratory (UCTEL) at the University of Manitoba. Concentrations of MeHg were measured on an automated MeHg analyzer (MERX-M, Brooks Rand) following an adapted ascorbic acid-assisted direct ethylation method^39^, which involves ethylation, Tenax trap pre-concentration, gas chromatographic separation and CVAFS quantification. The original method^39^ was modified for use with ~40-mL sample volumes using acetate buffers to adjust pH. Daily calibration curves were prepared by adding standards solutions to filtered seawater to improve recovery from the seawater matrix. The detection limit (DL) was estimated at 0.25 pM and 0.014 pM for Hg_T_ and MeHg, respectively, as three times the standard deviation of seven laboratory blank replicates. Whenever seawater was sampled, Milli-Q water was collected in pre-cleaned 250-mL amber glass bottles to serve as field blanks, the concentrations of which were always lower than the DL for both Hg_T_ and MeHg. Certified reference seawater BCR579 (9.5 ± 2.5 pmol kg^−1^ or 9.7 ± 2.5 pM when corrected for density, Institute for Reference Materials and Measurements, European Commission - Joint Research Centre) was analyzed for Hg_T_ and the recovery was 93–116%. Since no certified reference seawater is available for MeHg, a 0.01 pmol MMHg spike was used during sample analysis and its recovery was 87–114%.

Seawater fluorescence was measured in real-time with a chlorophyll fluorometer (Seapoint) installed on the rosette. To calibrate the fluorometer output, discrete seawater samples were measured for chlorophyll-α fluorescence concentrations.

## Electronic supplementary material

Supplementary Information

## Data Availability

The datasets generated during and/or analysed during the current study are available from the corresponding author on reasonable request.
