# Supplementary material for: Subsurface seawater methylmercury maximum explains biotic mercury concentrations in the Canadian Arctic
Source: Sci Rep. 2018 Sep 27;8:14465. doi: 10.1038/s41598-018-32760-0 (PMC6160454; doi:10.1038/s41598-018-32760-0)
Supplement: Supplementary file 1 — Supplementary Information [file 41598_2018_32760_MOESM1_ESM.pdf]

Supplementary Information

Subsurface seawater methylmercury maximum explains biotic mercury concentrations in the Canadian Arctic

Kang Wang[1], Kathleen M. Munson[1], Alexis Beaupré-Laperrière[2], Alfonso Mucci[2], Robie Macdonald[1,3], & Feiyue Wang[1*]

[1]Centre for Earth Observation Science, and Department of Environment and Geography, University of Manitoba, Winnipeg, Manitoba, R3T 2N2, Canada

[2]GEOTOP and Department of Earth and Planetary Sciences, McGill University, Montreal, Quebec, H3A 0E8, Canada

[3]Institute of Ocean Sciences, Department of Fisheries and Oceans, Sidney, British Columbia, V8L 4B2, Canada

This supplementary information includes the following:

- Table S1
- Figure S1
- Figure S2

Table S1. Detailed information of the stations sampled during the 2015 Canadian Arctic GEOTRACES campaign

Station	Latitude	Longitude	Water Depth (m)	Sampling Date
K1	56.124°N	53.377°W	3312	07/14/2015
BB1	66.856°N	59.058°W	1040	08/03/2015
BB2	72.753°N	67.001°W	2371	08/09/2015
BB3	71.409°N	68.598°W	1272	08/06/2015
CAA1	74.521°N	80.611°W	636	08/10/2015
CAA2	74.321°N	80.499°W	702	08/10/2015
CAA3	73.984°N	80.465°W	690	08/11/2015
CAA4	74.123°N	91.521°W	193	08/14/2015
CAA5	74.533°N	90.802°W	259	08/13/2015
CAA6	74.761°N	97.433°W	260	08/15/2015
CAA7	73.666°N	96.552°W	219	08/15/2015
CAA8	74.139°N	108.837°W	563	09/23/2015
CAA9	76.331°N	96.754°W	336	09/26/2015
CB1	75.118°N	120.628°W	466	09/07/2015
CB2	75.799°N	129.247°W	1365	09/08/2015
CB3	76.990°N	140.036°W	3731	09/12/2015
CB4	74.999°N	149.987°W	3830	09/16/2015

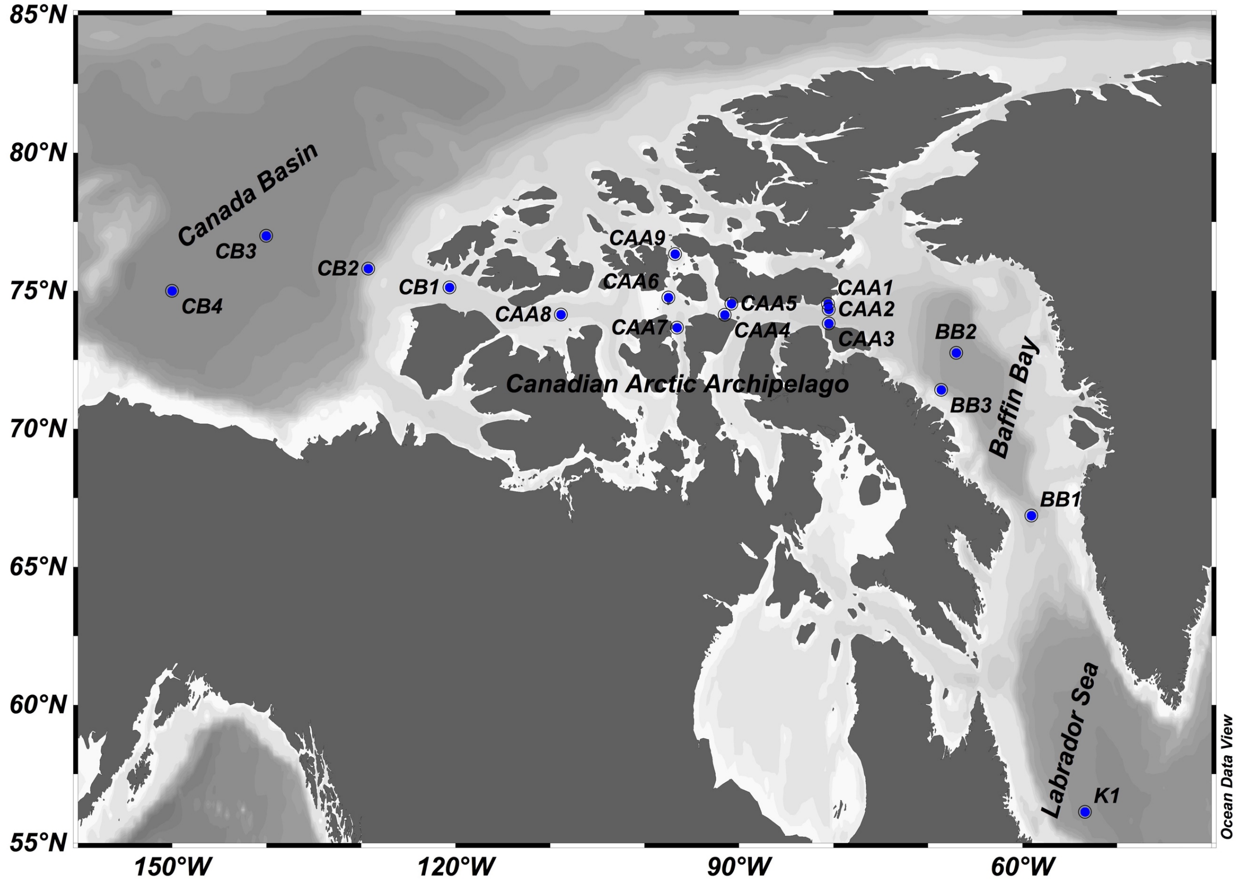

Figure S1. Location map of sampling stations during 2015 Canadian Arctic GEOTRACES Cruises. The base map with bathymetry was created using Ocean Data View (version 4.0; https://odv.awi.de).

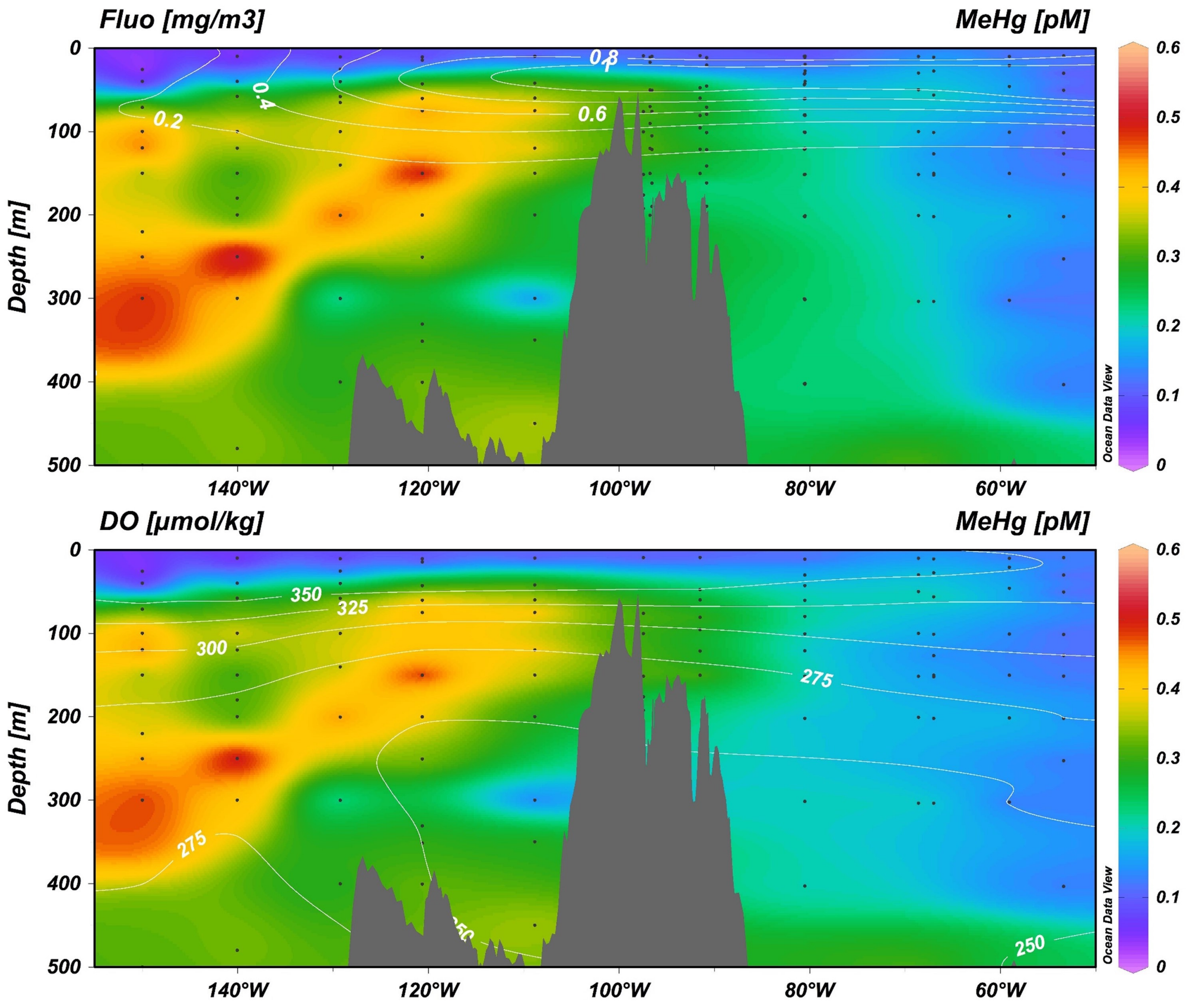

Figure S2. Distributions of methylmercury (MeHg, color coded) overlaid with the contours of the chlorophyll-α fluorescence (Fluo, top) and dissolved oxygen (DO, bottom) in the upper 500 m seawater across the Canadian Arctic and Labrador Sea. The base map with bathymetry was created using Ocean Data View (version 4.0)[1].

Reference:

1 Schlitzer, R. Ocean Data View, odv.awi.de (2018).
